# Enhanced Antioxidant Capacity and Anti-Ageing Biomarkers after Diet Micronutrient Supplementation

**DOI:** 10.3390/molecules190914794

**Published:** 2014-09-17

**Authors:** Aneta Balcerczyk, Agnieszka Gajewska, Ewa Macierzyńska-Piotrowska, Tomasz Pawelczyk, Grzegorz Bartosz, Janusz Szemraj

**Affiliations:** 1 Department of Molecular Biophysics, University of Lodz, Pomorska 141/143, Lodz 90-236, Poland; 2 Department of Affective and Psychotic Disorders, Medical University of Lodz, Czechoslowacka 8/10, Lodz 92-216, Poland; 3 Department of Medical Biochemistry, Medical University in Lodz, Mazowiecka 6/8, Lodz 92-215, Poland

**Keywords:** antioxidants, redox homeostasis, ageing, diet supplements

## Abstract

A growing number of studies confirm an important effect of diet, lifestyle and physical activity on health status, the ageing process and many metabolic disorders. This study focuses on the influence of a diet supplement, NucleVital^®^Q10 Complex, on parameters related to redox homeostasis and ageing. An experimental group of 66 healthy volunteer women aged 35–55 supplemented their diet for 12 weeks with the complex, which contained omega-3 acids (1350 mg/day), ubiquinone (300 mg/day), astaxanthin (15 mg/day), lycopene (45 mg/day), lutein palmitate (30 mg/day), zeaxanthine palmitate (6 mg/day), L-selenomethionine (330 mg/day), cholecalciferol (30 µg/day) and α-tocopherol (45 mg/day). We found that NucleVital^®^Q10 Complex supplementation significantly increased total antioxidant capacity of plasma and activity of erythrocyte superoxide dismutase, with slight effects on oxidative stress biomarkers in erythrocytes; MDA and 4-hydroxyalkene levels. Apart from the observed antioxidative effects, the tested supplement also showed anti-ageing activity. Analysis of expression of SIRT1 and 2 in PBMCs showed significant changes for both genes on a mRNA level. The level of telomerase was also increased by more than 25%, although the length of lymphocyte telomeres, determined by RT-PCR, remained unchanged. Our results demonstrate beneficial effects concerning the antioxidant potential of plasma as well as biomarkers related to ageing even after short term supplementation of diet with NucleVital^®^Q10 Complex.

## 1. Introduction

Ageing is an unavoidable, universal biological phenomenon affecting all multicellular organisms (with a few apparent exceptions). Various hypotheses have been put forward to explain the molecular reasons for ageing, a prominent role among them being played by the free radical theory of ageing proposed originally by Harman [1] and reformulated later on by various authors [2,3]. The redox potential of the O_2_/2H_2_O redox system (approximately +0.8 V at pH 7) is more positive than those of most other biologically relevant redox systems. Organic compounds and structures composed of them are thermodynamically unstable in an oxygen-containing atmosphere. Molecular oxygen in its basic triplet state is rather unreactive due to the spin restriction, but the formation of oxygen free radicals and other reactive oxygen species (ROS) opens the gate for potentially deleterious oxidative reactions of oxygen [4]. Thus, the free radical theory of ageing seems to address the very concept of the intrinsic biological instability of living systems. The basic idea of the free radical theory of ageing is that free radicals and other ROS, formed unavoidably in the course of metabolism and arising due to the action of various exogenous factors, damage biomolecules, and the accumulation of this damage is the cause of age-related diseases and ageing.

If the assumptions of free radical theory of ageing are true, it should be possible to slow down the process of ageing by intervention in the rate of generation of reactive oxygen species and/or their reactions with vital macromolecules, *i.a*. by administration of exogenous antioxidants. This straightforward conclusion should, however, take into account that: (i) cells and organisms tend to maintain redox equilibrium so long-term antioxidant supplementation may be not effective; and (ii) ROS are not only deleterious species, but also play a role in signaling pathways so drastic intervention in their level may sometimes be counterproductive.

Numerous studies have been conducted on the effects of supplementation with antioxidant vitamins, other natural and synthetic antioxidants and antioxidant-containing preparations on the ageing and lifespan of model organisms. Results of these studies were divergent and, as summarized in recent reviews, did not provide consistent data on the life-prolonging effects of supplementation with exogenous antioxidants [5,6,7].

However, apart from the obvious examination of lifespan prolongation by antioxidant administration throughout most of the lifetime (long-lasting experiments), another approach to study the anti-ageing effects of antioxidants consists of short-duration experiments, in which functional tests compare the status of experimental animals before and after supplementation. An experiment of this type consisted in administration of N-*tert*-butyl-α-phenylnitrone (PBN) to aged Mongolian gerbils for 2 weeks. Such a treatment reduced the amount of protein carbonyls in brain, augmented the activity of glutamine synthetase and decreased the number of errors in radial arm maze patrolling behavior, normalizing the values to those typical for young animals [8]. Similarly, relatively old mice (17.5 months) fed a high-CoQ diet (2.81 mg/g) for 15 weeks showed improved performance in Morris water maze tests and reduced protein oxidative damage [9]. The aim of the present study was to examine in healthy volunteers the effect of short-term supplementation with an antioxidant formula on the chosen parameters related to ageing and oxidative stress.

## 2. Results and Discussion

It has been frequently emphasized that supplementation with natural products containing more than one antioxidant is more effective than administration of a single one [10,11]. The reason for this effect is not clear, but a partial explanation may lie in the possibility of synergism between the various antioxidants present in natural products [12,13]. As shown by Podmore and co-workers, the micronutrient content may be a critical point for the observed phenomenon [14]. There are studies showing that supplementation of diet with a single antioxidant (vitamin C, vitamin E or β-carotene) exerts more harm than benefits [15,16,17]. Supplementing diets of healthy volunteers with a high dose of vitamin C (500 mg/day) had a detrimental effect, *viz*. prooxidative changes detectable as elevated levels of DNA damages in lymphocytes isolated from tested individuals [14]. A synergistic interaction between vitamin C and vitamin E was shown to provide efficient prevention against lipid peroxidation [18]. In another study, supplementation with vitamin C only failed to reduce oxidative DNA damage in smokers [19]. This may support the hypothesis about correlation of efficiency with the synergistic effects of antioxidants.

In the study now presented, a complex preparation containing several components was used. A capsule of NucleVital^®^Q10 Complex consists of several micronutrients including carotenoids, vitamins, selenium, ubiquinone and omega-3 acids. Participants in the presented research study were medically monitored in terms of general healthcare parameters. Blood morphology was analysed as well as creatinine, aminotransferase activity and lipid profile, including total cholesterol (TC), HDL, LDL and triglycerides (TG) (Table 1). Twelve weeks of supplementation did not affect any of the parameters indicated above, but revealed significant changes in blood parameters related to redox homeostasis and the ageing process.

**Table 1:** Selected cardiovascular risk factors after NucleVital^®^Q10 Complex intake. Values are presented as mean ± SD. TG = triglycerides; TC = total cholesterol; HDL = high density lipoprotein; LDL = low density lipoprotein; ASPAT = aspartate aminotransferase; ALAT = alanine aminotransferase; # *p* > 0.001, paired T-test.

Parameter	Week 0	Week 12
**TG [mg/dL]**	80.05 ± 30.46	83.27 ± 37.77
**TC [mg/dL]**	211.41 ± 34.87	208.09 ± 36.01
**HDL [mg/dL]**	72.72 ± 16.51	72.48 ± 17.31
**LDL [mg/dL]**	122.73 ± 29.49	118.99 ± 29.93
**ASPAT [U/L]**	19.62 ± 5.05	19.20 ± 6.32
**ALAT [U/L]**	18.15 ± 8.65	17.98 ± 13.06
**Creatinine [mg/dL]**	0.65 ± 0.10	0.71 ± 0.10
**Vit D [ng/mL] #**	15.61 ± 6.66	29.16 ± 8.59

### 2.1. Redox Homeostasis of Blood after NucleVital^®^Q10 Complex Intake

**Figure 1:**
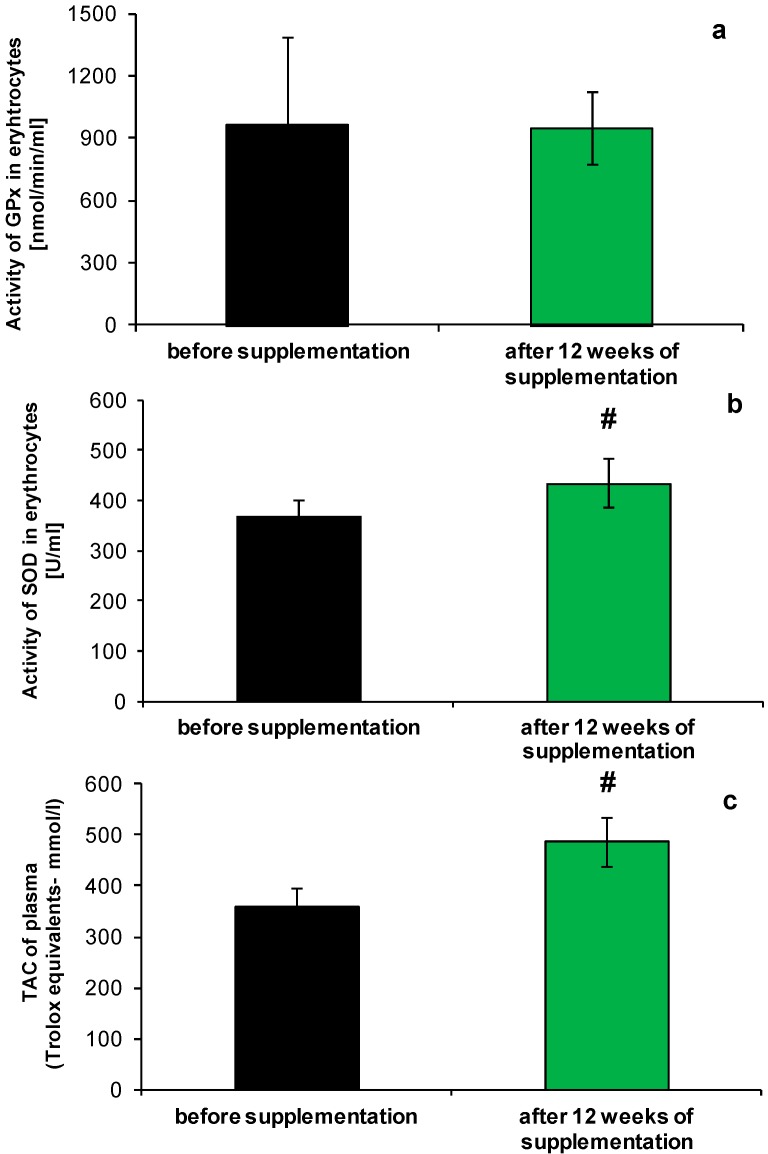
Changes in antioxidative potential of blood before and after NucleVital^®^Q10 Complex supplementation: (**a**) total antioxidant capacity of plasma (FRAP assay); (**b**) SOD activity; (**c**) GPx activity. # indicates statistically significant differences (*p* < 0.001).

We have found significant differences in the antioxidant capacity of plasma as measured with the FRAP assay, which reflects the sum of activities of plasma antioxidants, mainly uric acid, plasma protein thiol groups and ascorbic acid [20] (Figure 1a). An increase of about 25% was observed on the average, after the supplement intake. These results are in agreement with the reports of other authors, where the combined effect of multiple antioxidant supplementation on antioxidant status of healthy volunteers was studied [21,22]. Although the major component of the total antioxidant capacity of blood is uric acid, we assume that changes in total antioxidant capacity (TAC) may rather be related with α-tocopherol. This small antioxidant is present in the NucleVital^®^Q10 Complex and was delivered consistently by 12 weeks, but more detailed studies should be performed to reveal the background of the increased TAC.

Literature data show that TAC decreases significantly with age [23]. Our experiments performed in the presented study confirm this regularity. Reducing potential of plasma samples coming from 35–55 year old women, was significantly lower compared with that of women at the age of 25 (359.50 ± 35.41 *vs.* 548.06 ± 56.83 mmol Trolox equivalents/L). Twelve weeks of the tested micronutrient supplement intake, diminished the difference in FRAP values between both groups from 189.10 to 59.96 nmol/L of Trolox equivalents.

Analysis of selected elements of the enzymatic antioxidant system, revealed a slight increase of superoxide dismutase activity (SOD), by about 16%, in comparison to the state before the NucleVital^®^Q10 Complex supplementation (Figure 1b, *p* < 0.001). Enhanced ability of erythrocytes to scavenge superoxide anion did not extend to the removal of other reactive oxygen species (glutathione-dependent peroxidase (GPx) activity was unaffected by micronutrient supplement intake; Figure 1c). Increased SOD activity may be related with polyunsaturated fatty acids activity, present in the tested micronutrient preparation, as shown before [24,25], or the effect exerted by NucleVital^®^Q10 Complex on the SOD gene in pluripotent stem cells [26].

**Figure 2:**
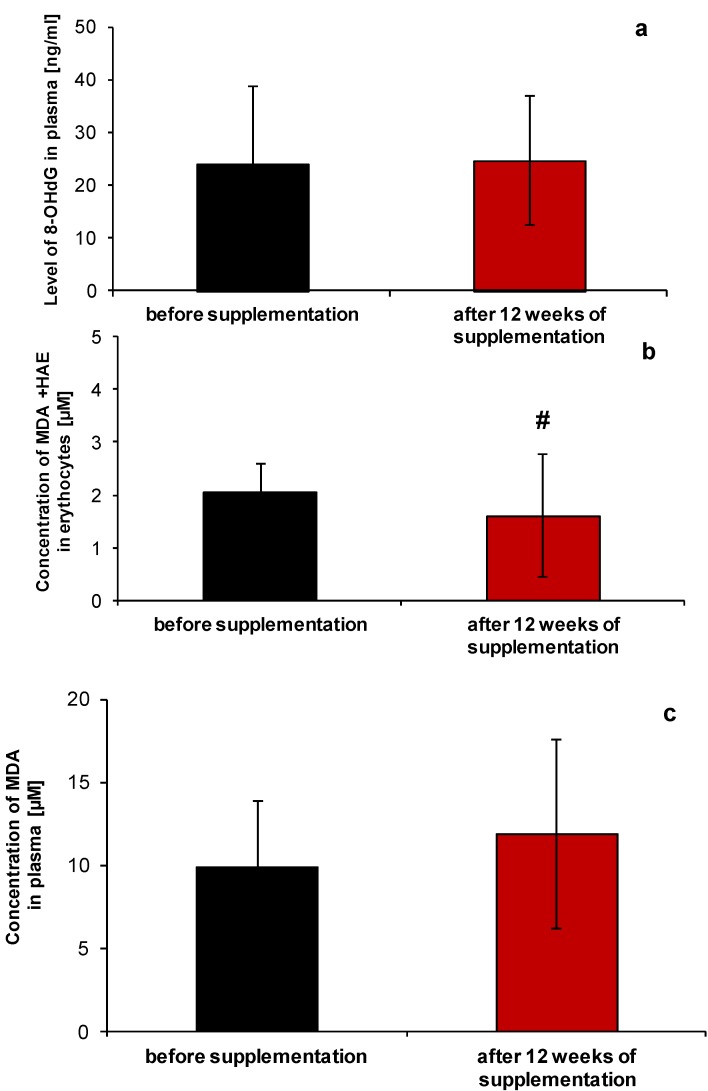
Oxidative stress biomarkers in blood samples of women before and after micronutrient supplementation with NucleVital^®^Q10 Complex; lipid peroxidation markers in erythrocytes (**a**), plasma (**b**) and level of 8-OHdG (**c**). # indicates statistically significant differences *p* < 0.02.

Analysis of other parameters involved in regulation of redox homeostasis showed that the combination of micronutrients present in the NucleVital^®^Q10 Complex significantly diminished lipid peroxidation in erythrocytes (Figure 2a; *p* < 0.02), while not affecting the lipid oxidation process in plasma (TBARS assay, Figure 2b). NucleVital^®^Q10 Complex treatment also did not affect the level of 8-OHdG (Figure 2c).

### 2.2. Anti-Ageing Abilities of NucleVital^®^Q10 Complex

**Figure 3:**
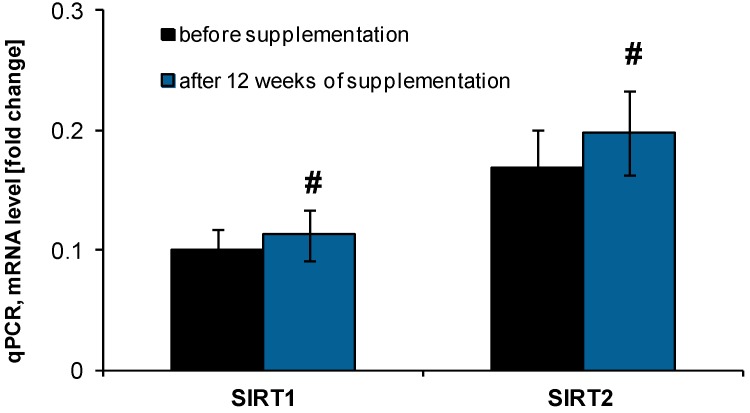
Expression of histone deacetylases (SIRT1 and SIRT2) in peripheral blood mononuclear cells isolated from blood of volunteers taking NucleVital^®^Q10 Complex. # indicates statistically significant differences (*p* < 0.001).

The revealed antioxidative abilities of the tested NucleVital^®^Q10 Complex led us to perform an analysis of parameters related to the ageing process, due to numerous studies indicating the role of antioxidants in limiting the ageing progress [27,28]. Using peripheral blood mononuclear cells (PBMCs) we have performed analysis of telomerase activity as well as expression of genes essential for senescence and regulation of oxidative stress (sirtuin 1 and 2) before and after 12 weeks of NucleVital^®^Q10 Complex intake. Sirtuins belong to class III histone deacetylases and are involved in regulation of inflammation, life/health span, calorie restriction/energetics, mitochondrial biogenesis, stress resistance, cellular senescence, endothelial functions, apoptosis/autophagy, and circadian rhythms through deacetylation of transcription factors and histones [29,30]. It is well known that SIRT1 expression as well as activity is decreased in chronic inflammatory states and ageing, conditions which are accompanied by increasing intensity of oxidative processes and the production of free radicals [29]. In our experimental conditions the performed gene expression analysis revealed a significantly increased level of both deacetylases (SIRT1 and SIRT2) of about 26.5% ± 12% and 25.0% ± 10%, respectively, after the micronutrient supplementation (Figure 3).

**Figure 4:**
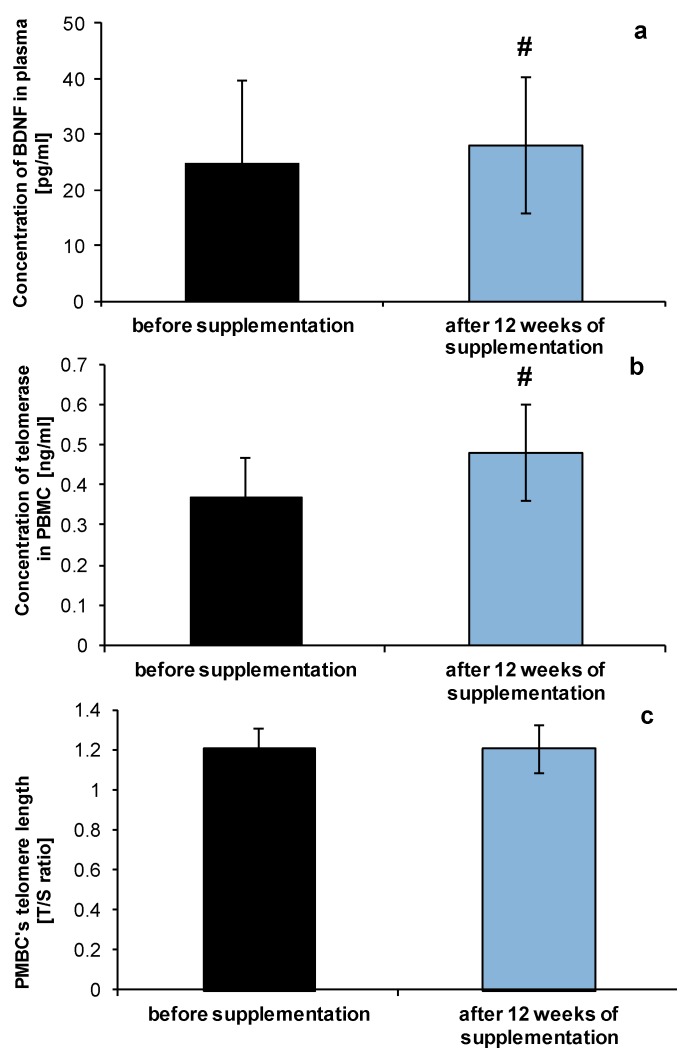
Level of BDNF (**a**), telomerase activity (**b**) and PMBC’s teleomere length (**c**) of healthy volunteers before and after NucleVital^®^Q10 Complex supplementation. # indicates statistically significant differences (*p* < 0.001).

The level of brain-derived neurotrophic factor (BDNF) was also elevated after 12 weeks of NucleVital^®^Q10 Complex diet supplementation (Figure 4a). The changes were estimated to be up to 26% ± 9% in comparison to control level (before the onset of supplementation). Decreased plasma level of BDNF is connected with memory and learning impairment [31] during the ageing process, in Alzheimer’s disease [32] and vascular dementia [33]. It would be interesting to check if the changes in analysed parameters are sustained despite lack of micronutrient intake and for how long.

On the other hand we have also found increased activity of telomerase (Figure 4b), which often serves as a potential biomarker of biological age [34], nevertheless PBMCs telomere length, was not changed (Figure 4c). Analysis performed by Cassidy and others [35] on the population of middle- and old-age women has shown the association between diet, and other lifestyle factors on leukocyte telomere length. Lack of changes in our experimental group may be related with supplementation with the tested micronutrient being too short-term. Maybe longer supplementation with the NucleVital^®^Q10 Complex would be more beneficial. Results of testing the effect of antioxidants on human health in other groups also showed that short-term supplementation might be beneficial in term of limiting the harmful effects of oxidative metabolism [36].

### 2.3. Effect of NucleVital^®^Q10 Complex on Vitamin D Deficiencies

A significant element in the tested micronutrient complex is vitamin D, present in an amount of 5 µg in a single capsule. As we have already mentioned all the volunteers were qualified for the study after a medical up to exclude any with pathological states, nevertheless average vitamin D levels in the plasma of recruited women, ranged around 15.61 ng/mL ± 6.66 ng/mL (Table 1) and was significantly below the minimum reference value, which is 30 ng/mL. Recent WHO data clearly show that about 70% of the European population displays suboptimal levels of vitamin D [37]. Vitamin D is essential for maintaining calcium and phosphate homeostasis. Severe and long-term deficiency in vitamin D levels can result in many metabolic disorders and diseases, such as osteoporosis or hyperparathyroidism [38]. Vitamin D modulates transcription of approximately 3% of the human genome via stimulation of vitamin D receptors. For example, renin, extracellular matrix metalloproteinases, and tumor necrosis factor-α are all regulated by vitamin D [39,40]. Both experimental and clinical studies support the health benefits of vitamin D, and its analogues, in the cardiovascular system, and such benefits include protecting cardiac function, lowering blood pressure, improving endothelial function, inhibiting oxidative stress, and reducing the activity of renin-angiotensin system [40,41]. Twelve weeks of supplementation with NucleVital^®^Q10 Complex was enough to significantly make up for the deficiencies in vitamin D level up to 29.16 ng/mL ± 8.59 ng/mL, *p* < 0.001, (Table 1), which points to the effectiveness of the tested preparation in minimizing the risks of disorders resulting from reduced levels of vitamin D.

## 3. Experimental Section

### 3.1. Material

One capsule of NucleVital^®^Q10 Complex (Scandinavian Laboratories Inc., Mt. Bethel, PA, USA; Marinex Intenational, Lodz, Poland) contains Norwegian salmon oil: omega-3 acids (225 mg; including 75 mg of eicosapentaenoic acid and 75 mg of docosahexaenoic acid), ubiquinone (50 mg) astaxanthin (2.5 mg), lycopene (7.5 mg), lutein palmitate (5 mg), zeaxanthin palmitate (1 mg), l-selenomethionine (55 mg), cholecalciferol (5 µg) and α-tocopherol (7.5 mg). The daily dose of NucleVital^®^Q10 Complex (six capsules per day) was calculated based on available literature data describing the beneficial effects of the indicated compounds on human health, the ageing process and improvement of energetic efficacy of the organism [42,43,44,45,46,47,48].

All experiments were performed on blood samples collected from healthy volunteers using citrate as an anticoagulant. For the particular assays plasma, erythrocytes and peripheral blood mononuclear cells were used. Erythrocytes were obtained by centrifugation of the blood samples (500× *g*, 10 min, 4 °C). After removing plasma and peripheral blood mononuclear cells, a pellet of erythrocytes was washed 3-times with ice-cold PBS buffer and hematocrit was measured. The suspension was adjusted to 60% hematocrit and this suspension was used in further analysis for preparation of lysates.

#### 3.1.1. Study Design

The presented study was carried out as a controlled supplementation study, focused on the effects of antioxidant micronutrients on selected parameters of redox homeostasis and ageing-related markers, and based on the Bioethical Committee agreement RNN/84/11/KE. The experimental group consisted of 66 women, 35–55 years old, who supplemented their diet with six capsules per day of NucleVital^®^Q10 Complex. The supplementation period lasted 12 weeks. Redox homeostasis and ageing-related parameters were determined in blood samples, before start of supplementation (week 0) and at week 12. To compare changes in antioxidant activity related with ageing (TAC assay, control/basic level), another group (*n* = 34) of 25 year-old women was recruited. Before the experiments, all the recruited participants were subjected to 4 weeks of washout. During the experiment, recruited women were not taking any other medicines or vitamin/dietary supplements.

#### 3.1.2. Inclusion/Exclusion Criteria

The inclusion criteria of women to the presented study were as follows: age 25–55 years, normal nutritional habits and being a non-smoker, non-vegetarian, and not pregnant or lactating, BMI 18.5–27, no intake of vitamin supplements, no history of liver diseases or lipid metabolism diseases, triglycerides <300 mg/dL, total cholesterol <300 mg/dL, sedimentation rate of erythrocytes <30 mm/h. Screening procedures checked the general health of the participants and included the medical history.

### 3.2. Analysis of Selected Parameters of Redox Homeostasis and Oxidative Stress

#### 3.2.1. Measurement of Superoxide Dismutase Activity

Measurement of total SOD activity (Cu/Zn-, Mn-, Fe-SOD) in erythrocytes was determined in diluted hemolysates using a colorimetric assay kit (Cayman Chemical Company, Ann Arbor, MI, USA), based on the ability of SOD to dismutation of superoxide anion generated during the xanthine/xanthine oxidase reaction, using a tetrazolium salt as an indicator. The assay was performed according to the instructions provided by the manufacturer.

#### 3.2.2. Measurement of Glutathione Peroxidase (GPx) Activity

Measurement of activity of glutathione peroxidase was determined in erythrocytes using a colorimetric assay kit (Cayman Chemical Company), based on oxidation of NADPH monitored by the rate of decrease in absorbance at 340 nm. Hemolysate was prepared by mixing erythrocytes with water, producing lysate corresponding to 12% hematocrit. Prepared samples were diluted 5-fold for estimation of enzyme activity. The assay was performed according to the provided manufacturer’s instructions.

#### 3.2.3. Total Antioxidant Capacity

Total antioxidant capacity of plasma samples was estimated by measuring of ferric reducing antioxidant power (FRAP) [49]. The assay is based on the ability of antioxidants to reduce of a ferric ion-2,4,6-tripyridyltriazine (TPTZ) complex to its ferrous form, which is monitored by the change in absorption at 593 nm. Briefly, plasma samples were diluted 30-fold with a FRAP reagent containing: (i) 300 mM acetate buffer, pH 3.6; (ii) 10 mM TPTZ in 40 mM HCl; (iii) 20 mM ferric chloride, mixed in a ratio of 10:1:1, respectively. Changes in absorbance were monitored for 4 min following the preincubation of samples at 37 °C. The results were expressed in Trolox equivalents.

#### 3.2.4. Lipid Peroxidation Assay

Measurement of lipid peroxidation levels was performed using colorimetric assay kits for plasma samples—TBARS assay kit (Cayman Chemical Company), and BIOXYTECH^®^LPO-586™ kit (OxisResearch™, Manhattan Beach, CA, USA) for erythrocytes. The assays are based on the measurement of malondialdehyde (MDA) and/or 4-hydroxyalkenes (HAE) generated due to a decomposition of polyunsaturated fatty acid peroxides. To stop the progress of lipid peroxidation in analysed samples during the assay procedure, butylated hydroxytoluene (BHT) was added to all samples and reagents, to a final concentration of 5 mM. The MDA level (plasma samples) and MDA + HAE level (erythrocytes) assays were performed according to the manufacturers’ instructions.

#### 3.2.5. Measurement of 8-OHdG Level

To determine the level of 8-OHdG in plasma samples, an enzyme-like immunosorbent assay (BioVendor, Brno, Czech Republic) was used. To remove all interfering substances, filtration of plasma samples was performed. The assay was performed according to the manufacturer’s instructions.

### 3.3. Analysis of Ageing-Related Parameters

#### 3.3.1. RNA Isolation and cDNA Synthesis

Total RNA was isolated from 200 μL of whole blood samples using a Norgen Leukocyte RNA Purification Kit (Norgen Biotek Corporation, Thorold, ON, Canada), according to the provided protocol. Purified RNA was used as a template for RT-qPCR reaction. Concentration of RNA was measured with a Nanodrop system (Thermo Scientific, Wilmington, DE, USA) and 2 µg of DNA-free RNA was converted to cDNA using SuperScript Reverse Transcriptase III and random hexamers (Invitrogen, Carlsbad, CA, USA).

#### 3.3.2. Estimation of SIRT1 and SIRT2 Expression

PCR amplification was performed using an ABI Prism 7900 Sequence Detection System (Applied Biosystems, Carlsbad, CA, USA). Forward and reverse primer (0.1 µM of each)m, cDNA template and SYBRGreen Master Mix (Applied Biosystems) were mixed to a final volume of 20 µL. Reactions were incubated at 95 °C for 5 min, followed by 40 cycles of 95 °C for 15 s and 60 °C for 30 s. Analyzed genes were normalized to the level of 18S. All the primer sequences (18S: 5'-CCGATAACGAACGAGACTCTGG-3', 5'-TAGGGTAGGCACACGCTGAGCC-3'; SIRT1: 5'-TACCGAGATAACCTTCTGTTCG-3', 5'-GTTCGAGGATCTGTGCCAAT-3'; SIRT2: 5'-AGAAGCAGACATGGACTTCCT-3', 5'-CTCCCACCAAACAGATGAC-3') were obtained from Qiagen Inc., Valencia, CA, USA. Each sample was analyzed in triplicate. Real-Time PCR data were automatically calculated with the data analysis module. The results were analyzed according to the 2^−^^Δ^^ΔCt^ method. Validation of PCR efficiency was performed with a standard curve.

#### 3.3.3. Measurement of BDNF Level

Measurement of brain-derived neurotrophic factor (BDNF) level was determined in plasma samples using a Quantikine ELISA Human BDNF ELISA kit (R&D Systems, Minneapolis, MN, USA), by measurement of absorbance at 450 nm with correction at 540 nm. The assay was performed according to the manufacturer’s instructions.

#### 3.3.4. Measurement of Telomerase Level

Measurement of telomerase level was determined in peripheral blood mononuclear cells using a Human ELISA Telomerase kit (Biocompare, San Francisco, CA, USA), by measurement of absorbance at 450 nm with correction at 540 nm. The assay was performed according to the manufacturer’s instructions.

#### 3.3.5. Measurement of PBMC’s Telomere Length

A quantitative real-time polymerase reaction method [50] was used to measure relative telomere length in genomic DNA extracted from peripheral blood mononuclear cells. PCR reactions were set up by aliquoting 15 μL of master mix with final concentration of reagents in the PCR reaction: 20 ng of DNA, 0.75 × SYBR Green I (Invitrogen), 10 mM Tris-HCl MgCl_2_ pH 8.3, 50 mM KCl, 3 mM MgCl_2_, 0.2 mM dNTP, 1 mM DTT, 1 mM betaine (Sigma, Saint-Louis, MO, USA) and 0.5 U AmpliTaq Gold DNA polymerase (Applied Biosystems, Inc.). For multiplex QPCR, the telomere pair telg: 5'-ACACTAAGGTTTGGGTTTGGGTTTGGGTTTGGGTTAGTGT-3', telc: 5'-TGTTTAGGTATCCCT ATCCCTATCCCTATCCCTATCCCTAACA-3', (final concentration of each primer 900 nM) were combined with albumin primer pair albu: 5'-CGGCGGCGGGCGGCGCGGGCTGGGCGG aaatgctgccacagaatccttg-3' and albd: 5'-GCCCGGCCCGCCGCGCCCGTCCCGCCGgaaaagcatggtcg cctgtt-3' (final concentration of each primer 500 nM). The thermal cycling profile was: 15 min 95 °C, 2 cycles of 15 s at 94 °C, 15 s at 49 °C, and 32 cycles of 15 s at 94 °C, 10 s at 62 °C, 15 s at 74 °C with signal acquisition 10 s at 84 °C, 15 s at 88 °C. The data are expressed as T/S ratio, where (T) is the number of nanograms of the standard DNA that matches the experimental sample for copy number of the telomere template, and (S), the number of nanograms of the standard DNA that matches the experimental sample for copy number of scg. Each experimental sample was assayed in triplicate, and the final reported result for a sample is the average of three T/S values. Average T/S expected to be proportional to the average telomere length per sample. Samples with a T/S > 1.0 have average telomere length greater than that of standard DNA; samples with T/S < 1.0 have average telomere length shorter than that of standard DNA.

### 3.4. Measurement of Vitamin D Level

Determination total 25-OH vitamin D level was performed using a commercially available chemiluminescent immunoassay (CLIA) kit LIAISON 25-OH Vitamin D total Assay (DiaSorin Inc., Stillwater, MN, USA) for plasma samples.

### 3.5. Statistical Analysis

The results of the investigation are expressed as means ± SD. Statistical significance of changes in analyzed parameters, before and after taking the NucleVital^®^Q10 Complex, was done using the paired *t*-test. Values of *p* < 0.05 were considered as significant.

## 4. Conclusions

In conclusion, the performed analyses of selected parameters related to oxidative stress and ageing process on a population of middle and older-age women, allow us to conclude that short term diet supplementation with NucleVital^®^Q10 Complex, being a mixture of antioxidants, vitamins and selenium, seems to beneficial and can be recommended. The tested micronutrient mix significantly makes up deficiencies in vitamin D level, reduces markers of lipid peroxidation, and at the same time increases antioxidant potential of plasma as well as affects positively markers related to the ageing process.
